# A case of combined hamartoma of the retina and retinal pigment epithelium with response to intravitreal ganciclovir injection

**DOI:** 10.5935/0004-2749.20220094

**Published:** 2025-08-21

**Authors:** Seo-Yeon Hong, Jae Hoon Lee, Mee Yon Lee

**Affiliations:** 1 Department of Ophthalmology, College of Medicine, The Catholic University of Korea, Seoul, Republic of Korea; 2 Department of Ophthalmology, Uijeongbu St. Mary’s Hospital, College of Medicine, The Catholic University of Korea, Uijeongbu-si, Republic of Korea

Dear Editor,

Combined hamartoma of the retina and retinal pigment epithelium (CHRRPE) is a relatively uncommon benign, elevated, pigmented lesion involving the retina, retinal pigment epithelium, blood vessels, and vitreous^(1)^. It is commonly associated with vitreoretinal interface changes and often leads to retinal traction or epiretinal membrane formation^(2)^. CHRRPE presents in isolated forms, and among them, cases associated with systemic diseases, such as neurofibromatosis or brachio-oto-renal syndrome, have been reported, but their etiology remains uncertain^(3)^. Although the option of surgery for the management of CHRRPE remains controversial, thus far, epiretinal membrane surgery is the only treatment method that has been attempted, and no other treatment options are established^(4)^. This paper describes a case of CHRRPE that was initially misdiagnosed with cytomegalovirus (CMV) retinitis but incidentally showed a good response to intravitreal ganciclovir injection.

**Figure 1:**
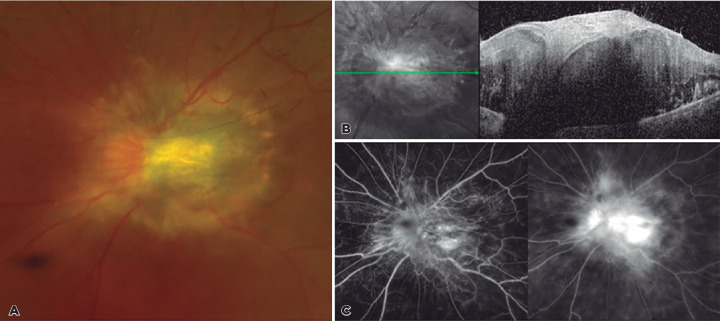
Fundus photography (A), OCT imaging (B), and fluorescein angiography (C) of combined hamartoma of the retina and retinal pigment epithelium (CHRRPE) in a 31-year-old male at presentation.

A 31-year-old male presented to our clinic with a history of blurred vision in the left eye since the previous 1 month. He was referred with atypical CMV retinitis, with seropositive immunoglobulin G and M antibodies against CMV. Visual acuity of the left eye was finger counting from 50 cm and noncorrectable. Results of the anterior segment examination were within normal limits. Fundus examination demonstrated a grayish elevated mass covering the optic disc with surrounding tortuous vessels and capillary telangiectasia. Optical coherence tomography (OCT) revealed a hyper-reflective lesion at the level of inner retina with hypo-reflective shadowing posteriorly, leading to obscuration of the underlying retinal structures discrete from the adjacent unaffected retinal layers. Fluorescein angiography highlighted irregular dilations of the retinal capillary network leading to leakage of the fluorescein dye, with no evidence of choroidal neovascularization (Figure 1).

**Figure 2:**
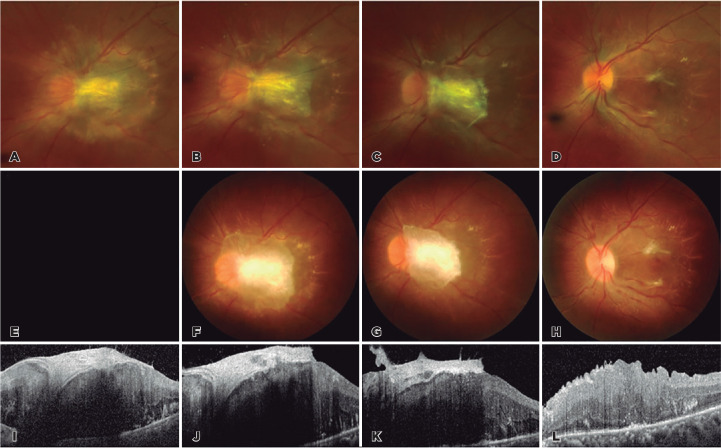
Regression of CHRRPE in response to intravitreal ganciclovir injection and after pars plana vitrectomy. Fundus photography, disc stereophotography, and OCT imaging at presentation (A, E, I), after two injections (B, F, J) and six injections (C, J, K) demonstrate the decreasing size of the mass and fibrovascular membrane. At 1 week after pars plana vitrectomy (D, H, L), the optic disc margin appears clear with dimpling and distortion of the retinal layers.

Polymerase chain reaction analysis of CMV on the aqueous humor was negative, and the patient had no evidence of immunosuppressive status, with negative human immunodeficiency virus results. Differential diagnosis of retinal vascular proliferation on Von Hippel Lindau syndrome was also considered, but there was no evidence of coherent hemangioblastomas in other organs such as brain, kidney, or pancreas, with negative genetic testing results. No other differential diagnosis could be made at that time, and intravitreal ganciclovir injection was attempted with a diagnosis of atypical CMV retinitis. After two injections of intravitreal ganciclovir, the nasal margin of the optic disc clearly appeared, and the mass seemed to be regressed, especially on the temporal side. As the intravitreal ganciclovir injection was repeated, the mass continued to decrease in size and optic disc margin became clearer on the fundoscopy. Moreover, the fibrovascular membrane on the mass became distinguishable on the OCT. After six injections of intravitreal ganciclovir, the mass decreased in size and fibrovascular membrane markedly regressed, but visual acuity in the left eye remained finger counting from 50 cm (Figure 2). Pars plana vitrectomy was then performed with membrane peeling. By 1 week postoperatively, components of the fibrovascular membrane were completely removed with increased integrity of the macular morphology, as identified by fundoscopy and OCT (Figure 2). Visual acuity also improved to 0.02 Snellen at 1 month postoperatively.

CHRRPE are a peculiar pigmented, elevated mass that is considered to be benign but can cause significant loss of vision associated with macular distortion^(5)^. Thus far, the etiology and management of CHRRPE remains controversial due to few cases and sporadic studies. This case was a typical case of CHRRPE that incidentally responded to intravitreal ganciclovir injection. Several presumptions could be made regarding the response of this CHRRPE case to intravitreal ganciclovir injection. First, the natural progression of membrane contraction of CHRRPE could have happened by chance. Second, intravitreal ganciclovir injection may have induced vitreous changes that led to membrane contraction. Finally, CHRRPE could be associated with viral factors that are not yet known. Prior studies on CHRRPE focused on its clinical manifestations and morphologic features, especially on the OCT^(2,3)^. A few studies reported CHRRPE associated with systemic diseases, such as neurofibromatosis, and recent studies focused on the efficacy of surgical management^(4,5)^. However, CHRRPE remains a rare benign tumor that can cause serious visual deterioration with uncertain etiology and controversial treatment options. Therefore, this CHRRPE case with response to intravitreal ganciclovir injection may be helpful to future investigations on the pathophysiological and therapeutic approaches for CHRRPE.
